# Common principles and intermediates of viral protein-mediated fusion: the HIV-1 paradigm

**DOI:** 10.1186/1742-4690-5-111

**Published:** 2008-12-10

**Authors:** Gregory B Melikyan

**Affiliations:** 1 Institute of Human Virology, Department of Microbiology and Immunology, University of Maryland School of Medicine, 725 W. Lombard St, Baltimore, MD 21201, USA

## Abstract

Enveloped viruses encode specialized fusion proteins which promote the merger of viral and cell membranes, permitting the cytosolic release of the viral cores. Understanding the molecular details of this process is essential for antiviral strategies. Recent structural studies revealed a stunning diversity of viral fusion proteins in their native state. In spite of this diversity, the post-fusion structures of these proteins share a common trimeric hairpin motif in which the amino- and carboxy-terminal hydrophobic domains are positioned at the same end of a rod-shaped molecule. The converging hairpin motif, along with biochemical and functional data, implies that disparate viral proteins promote membrane merger via a universal "cast-and-fold" mechanism. According to this model, fusion proteins first anchor themselves to the target membrane through their hydrophobic segments and then fold back, bringing the viral and cellular membranes together and forcing their merger. However, the pathways of protein refolding and the mechanism by which this refolding is coupled to membrane rearrangements are still not understood. The availability of specific inhibitors targeting distinct steps of HIV-1 entry permitted the identification of key conformational states of its envelope glycoprotein *en route *to fusion. These studies provided functional evidence for the direct engagement of the target membrane by HIV-1 envelope glycoprotein prior to fusion and revealed the role of partially folded pre-hairpin conformations in promoting the pore formation.

## Review

Enveloped viruses initiate infection by fusing their membrane with the cell membrane and thereby depositing their genome into the cytosol. This membrane merger is catalyzed by specialized viral proteins referred to as fusion proteins. When activated via interactions with cellular receptors and/or by acidic endosomal pH, these proteins promote membrane merger by undergoing complex conformational changes (reviewed in [1,2]). The principal challenges facing researchers studying molecular details of this process are: (i) limited structural information about fusion proteins and their refolding pathways; (ii) transient and generally irreversible nature of conformational changes; and (iii) often redundant number of proteins the majority of which may undergo off-pathway refolding. In spite of these obstacles, considerable progress has been made towards understanding viral fusion, as discussed in a number of excellent reviews [1-6]. The emerging picture is that disparate enveloped viruses have adapted a common strategy to fuse membranes. This review will discuss the general principles by which viral proteins promote fusion, focusing on the retroviral envelope (Env) glycoproteins exemplified by HIV-1 Env.

## Intermediates of lipid bilayer fusion

**Figure 1:**
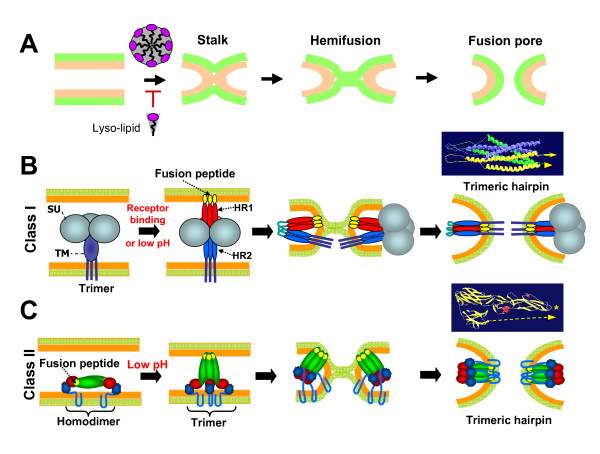
**The stalk-pore model of lipid bilayer fusion**. (A) and consensus models for class I and class II protein-mediated membrane fusion (B and C). SU and TM are the surface and transmembrane subunits of a fusion protein, respectively. Fusion peptides/domains are colored yellow. The structure in B is the trimeric core of the Simian Immunodeficiency Virus gp41 in a post-fusion conformation. The yellow triangle and arrow represent the position and orientation of the membrane spanning domain and the fusion peptide, respectively. The structure in C is the Dengue Virus E protein fragment in its post-fusion conformation (a monomer is shown for visual clarity). The yellow dashed line and triangle represent the viral membrane-proximal segment and the membrane spanning domain, respectively. Asterisk marks the location of the fusion domain.

Whereas viral proteins regulate and promote the merger of biological membranes, complete fusion occurs when lipids from two distinct bilayers rearrange to form a continuous membrane. Thus, to elucidate the principles of protein-mediated fusion, it is essential to understand the mechanism of lipid bilayer fusion. The most prominent model for membrane fusion (Fig. 1A), referred to as the "stalk-pore" model [7], posits that contacting monolayers of two membranes are initially joined via a local saddle-shaped connection referred to as a "stalk" [8,9]. Lateral expansion of the lipid stalk permits the distal monolayers to come into direct contact and form a shared hemifusion diaphragm. Accumulated evidence suggests that hemifusion is a common intermediate in a variety of protein-mediated fusion reactions (for review, see [10]). The subsequent rupture of a hemifusion diaphragm results in the formation of a fusion pore through which both membrane and content markers redistribute [11,12].

## The structure-based classification of viral fusion proteins

Generally, fusion proteins of enveloped viruses are type I integral membrane proteins expressed as trimers or dimers [1-3,5,6]. With a few exceptions, these proteins are rendered fusion-competent upon post-translational cleavage by cellular proteases of either the protein itself or of an associated regulatory protein [1,2,13]. A salient feature of viral proteins is a highly conserved, functionally important stretch of hydrophobic residues referred to as the fusion peptide or the fusion domain [1,13,14]. In their mature, proteolytically cleaved form viral fusion proteins are thought to exist in a meta-stable, "spring-loaded" conformation [15], capable of releasing the energy as they transition to final conformation. While it is likely that this conformational energy drives fusion, the exact mechanism of coupling between protein refolding and membrane rearrangements is not fully understood.

Based on the structure of extracellular domains, viral fusion proteins are currently categorized into three classes. Fusion proteins of retroviruses, filoviruses, coronaviruses, ortho- and paramyxoviruses displaying a prevalent α-helical motif belong to the class I proteins [1,16,17]. In an initial conformation, the N-terminal or N-proximal hydrophobic fusion peptides of the TM subunit (Fig. 1B) are usually sequestered at the trimer interface. Perhaps the best studied representatives of the class I proteins are influenza hemagglutinin and HIV-1 envelope (Env) glycoprotein (reviewed in [18,19]). The defining feature of the class II fusion proteins of flaviviruses and togaviruses is the predominant β-sheet motif [1,3]. These fusogens are expressed as homo-dimers (tick-borne encephalitis virus E protein) or hetero-dimers (Semliki Forest Virus E1/E2 proteins) with their hydrophobic fusion domains sequestered from solution at the dimer interface (Fig. 1C). The newly identified class III viral proteins (rhabdoviruses and herpesviruses) exhibit both α-helical and β-sheet elements and thus appear to combine the structural features of first two classes [1,5,6]. Interestingly, fusion proteins of rhabdoviruses exemplified by the G protein of Vesicular Stomatitis Virus (VSV) undergo low pH-dependent transition from a pre-fusion to a post-fusion form, but, unlike other viral proteins, return to their initial conformation at neutral pH [20,21]. This unique reversibility implies that the difference in free energy of pre- and post-fusion conformations of G proteins is relatively small. Thus, the pre-fusion structure of this protein may not be viewed as meta-stable, suggesting that the "spring-loaded" mechanism [15] that relies on large changes in the protein's free energy may not be operational here [20].

## Model systems for studying viral fusion

While the structures of ectodomains (or their core fragments) have been solved for several viral proteins, information regarding intermediate conformations of full-length viral proteins in the context of fusing membranes is not available. Complementary functional assays are thus important for gaining insight into the refolding pathways of viral proteins. Mechanistic studies of viral fusion have been primarily carried out using a cell-cell fusion model [11,22,23]. Cell-cell fusion assays adequately reflect the activity of viral proteins, especially when early manifestations of fusion, such as small pore formation, are being monitored. Further, this model is ideally suited for manipulating experimental conditions and for convenient and reliable quantification of fusion products. However, there is increasing awareness of the fact that not all features of virus-cell fusion can be faithfully reproduced in this model. For instance, murine leukemia virus (MLV) undergoes receptor-mediated translocation ("surfing") along microvilli to a cell body before fusing to a plasma membrane [24]. An example of cellular compartment-specific entry is Ebola virus fusion that occurs after the cleavage of its glycoprotein by the lysosome-resident cathepsin B [25,26]. This intracellular activation of the fusion protein makes the cell-cell fusion model unsuitable for functional studies. The use of cell-cell fusion assays is also limited when surface expression of viral fusion proteins is low due to an endoplasmic reticulum retention signal. Examples of such glycoproteins include the Dengue Virus E [27] and Hepatitis C Virus E1/E2 [28] glycoproteins.

Until recently, direct techniques to measure virus-cell fusion were not available, and most functional studies employed infectivity assays to evaluate fusion [29-32]. However, measuring the levels of infection that rely on successful completion of viral replication steps downstream of fusion may underestimate the efficacy of fusion [33,34]. Novel techniques monitoring the delivery of viral core-associated enzyme into a host cell permit direct assessment of the extent and kinetics of virus-cell fusion [33-37], but these assays have limited sensitivity and temporal resolution. A powerful approach to study virus-cell fusion that circumvents fundamental limitations imposed by the heterogeneity of virus population is time-resolved imaging of single viral particles (e.g., [38-43]). Using this technique, important advances have been made towards understanding the mechanisms of receptor-mediated virus uptake, endosomal sorting, and towards identifying the preferred sites of virus entry [44-47]. Time-resolved imaging of viral lipid and content redistribution permitted visualization of intermediate steps of fusion between single HIV-1 and Avian Sarcoma and Leukosis Virus (ASLV) particles and target cells [48,49].

## Entry pathways and modes of activation

Viral proteins are activated through various mechanisms principally determined by the virus entry pathway [1,22,39,41,50]. Viruses that do not rely on low pH for entry are activated by binding to their cognate receptor(s) [51,52] and are thought to fuse directly with a plasma membrane. Fusion proteins of viruses entering cells via an endocytic pathway are mainly triggered by acidic pH in endosomes [1,39]. These viruses often use cellular receptors as attachment factors to facilitate their internalization. Interestingly, ASLV Env is activated via the two-step mechanism that involves binding the cognate receptor that renders Env competent to undergo conformational changes upon subsequent exposure to low pH in endosomes [53-59]. The two-step activation of viral fusogens is not uncommon. HIV Env is rendered fusogenic through sequential interactions with CD4 and a coreceptor [51,60]. Following receptor-mediated endocytosis, the Ebola virus glycoprotein is activated by proteolytic cleavage in lysosomes [25,26]. These multiple triggering steps may help sequester the conserved functional domains of viral fusion proteins from immune surveillance and/or ensure the release of the viral genome at preferred cellular sites.

## A generalized mechanism of viral fusion

In spite of structural differences, different classes of fusion proteins appear to promote membrane merger through a common "cast-and-fold" mechanism (reviewed in [1-6,11,16,22,23,61]). The critical evidence supporting this universal fusion mechanism is the conserved trimeric hairpin (or 6-helix bundle, 6HB) motif shared by post-fusion conformations of disparate viral proteins [1,6,16,17]. For class I fusion proteins, this structure is formed by antiparallel assembly of the central N-terminal trimeric coiled coil (or heptad repeat 1, HR1 domain) and three peripheral C-terminal helices (HR2 domains), as depicted in Fig. 1B. The antiparallel orientation of the C-terminal and N-terminal segments of ectodomains of class II and III viral proteins indicates that these proteins also form trimeric hairpin structures (Fig. 1C). An important implication of a hairpin structure is that, in the final conformation, the membrane-spanning domains (MSDs) and the hydrophobic fusion peptides, which are not a part of crystal structure, are positioned close to each other.

The following consensus model for viral protein-mediated fusion has emerged from the implicit proximity of the MSDs and fusion peptides in the conserved hairpin structures and from extensive biochemical and functional data (Fig. 1B, C). When triggered by receptor binding and/or by low pH, viral proteins insert their fusion peptides into a target membrane [62-66]. At this point, the initially dimeric class II proteins convert to fusion-competent homotrimers [3,6,13]. In addition to anchoring the viral proteins to the target membrane, the fusion peptides appear to destabilize lipid bilayers by promoting the formation of non-lamellar structures [14,67-69]. Next, the extended trimeric conformation bridging the viral and target membranes drives membrane merger by folding back on itself and forming a hairpin structure. Several lines of genetic and functional evidence support this model. First, mutations in the conserved fusion peptides [70-77] and those destabilizing the trimeric hairpin [78-82] attenuate or abrogate fusion. Second, peptides derived from the HR1 and HR2 regions of class I proteins (referred to as C- and N-peptides, respectively) inhibit fusion by binding to their complementary domains on the fusion protein and preventing 6HB formation (reviewed in [16]). Likewise, soluble fragments of class II fusogens also block fusion [83], apparently by preventing the formation of trimeric hairpins.

The general principles by which viral proteins cause membrane fusion are likely dictated by the physical properties of lipid bilayers which must form highly curved and thus energetically unfavorable intermediate structures (e.g., a stalk and a fusion pore). Accumulating evidence that fusion induced by distinct classes of viral proteins converges to a common hemifusion intermediate [49,56,84-89] further supports the universal mechanism of membrane merger.

While it is widely accepted that the transition from an initial conformation to a final hairpin drives fusion, the refolding pathways of viral proteins are poorly characterized. In discussing the conformational intermediates of class I viral proteins, this review will focus primarily on fusion induced by HIV-1 Env. Numerous antibodies to HIV-1 Env and entry inhibitors targeting the receptor binding and fusion steps are available for mechanistic studies of Env-mediated fusion. Recent functional work using various HIV fusion inhibitors provided new clues regarding the HIV entry process.

## Conformational changes of class I proteins: Lessons from HIV-1 Env-induced fusion

### Receptor binding and conformational changes in HIV-1 gp120 subunit

The transmembrane, gp41, and surface, gp120, subunits of HIV Env are generated upon cleavage of the gp160 precursor by furin-like proteases. Mature HIV Env is rendered fusogenic upon sequential interactions of gp120 with CD4 and coreceptors, CCR5 or CXCR4 [16,18,51,90]. Binding to CD4 alters the structure and conformational flexibility of gp120 resulting in formation of the coreceptor binding site that permits assembly of ternary gp120-CD4-coreceptor complexes [91-97]. Interestingly, Env glycoproteins from HIV-2 strains tend to undergo CD4-induced conformational changes and engage coreceptors much faster than HIV-1 Env [98]. The assembly of ternary complexes, in turn, triggers gp41 conformational changes culminating in formation of 6HBs in which the HR2 domains are packed in antiparallel orientation against the trimeric HR1 coiled coil (e.g., [16,17]).

The minimum number of CD4 and coreceptor molecules per Env trimer required to trigger fusogenic conformational changes has not been unambiguously determined [99-101]. Analysis of infection as a function of coreceptor density indicates that recruitment of 4–6 mutant CCR5 with attenuated affinity to gp120 per virion leads to infection [102]. On the other hand, the follow-up study using cells expressing CD4 and wild-type CCR5 concluded that recruitment of just one CCR5 molecule by CD4-bound Env could mediate infection [103]. However, clustering of HIV receptors within the membrane domains and modulation of HIV entry/fusion by homo-dimerization of CD4 and coreceptors [104,105] confound the determination of the requisite number of these molecules in a fusion complex. Recent evidence suggests that, in addition to CD4 and coreceptors, proteins catalyzing the thiol/disulfide exchange reaction play a role in triggering productive conformational changes in HIV-1 Env [106-109].

Little is known about the mechanism by which the formation of gp120-CD4-coreceptor complexes triggers refolding of gp41. The notion that gp120 has to detach from gp41 (termed gp120 shedding) in order to lift the restriction on gp41 refolding is a subject of debate [110-114]. While the relevance of complete gp120 shedding to fusion has not been convincingly demonstrated, there is evidence that interactions between gp120 and gp41 must weaken in order to initiate fusion [115]. Introduction of a disulfide bond between non-covalently associated gp120 and gp41 subunits rendered Env inactive. However, this mutant could be re-activated by reducing the disulfide bond after allowing the Env to interact with CD4 and coreceptors on target cells. Under these conditions, reduction-induced fusion was resistant to coreceptor binding inhibitors, implying that the receptor/coreceptor binding function was not compromised by linking gp120 and gp41 subunits [115]. These findings suggest that, following the formation of ternary complexes with CD4 and coreceptor, gp120 must, at least partially, disengage gp41 to permit the fusogenic restructuring of the latter subunit.

### HIV-1 gp41 refolding

Two complementary approaches have been employed to follow the progression of gp41 through intermediate conformations. The formation/exposure of novel gp41 epitopes has been assessed via antibody reactivity using an immunofluorescence assay or by measuring the binding of gp41-derived peptides to their complementary HR1/HR2 domains [116-119]. Alternatively, the exposure of the HR1 and HR2 domains has been indirectly detected based on the ability of gp41-derived inhibitory peptides to block the progression to full fusion after these peptides were introduced and washed off at an arrested intermediate stage [120-124] (see below). A set of gp41 conformations on which the HR1 and/or HR2 domains are exposed will hereafter be referred as pre-bundles [123].

#### Exposure of gp41 epitopes

Immunofluorescence experiments demonstrated that the gp41 HR1, as well as the immunogenic cluster I (residues 598–604) and cluster II (residues 644–663) overlapping the gp41 loop and HR2 domain, respectively, are transiently exposed during fusion [116-118]. The HR1, HR2 and loop domains become available as early as upon CD4 binding and are lost concomitant with the onset of cell-cell fusion. By comparison, the tryptophan-rich membrane-proximal external region (MPER), which is C-terminal to the gp41 HR2 domain, is accessible to the neutralizing antibodies, 2F5 and 4E10, on the native structure, but the MPER accessibility is gradually lost as fusion progresses to the content mixing stage [116,117,125]. The exposure of HR1 and HR2 domains upon interactions with CD4 is also supported by the enhanced binding of C- and N-peptides targeting these domains [117,119,126-128]. To conclude, gp120-CD4 and gp120-coreceptor interactions reportedly result in (at least transient) exposure of HR1 and HR2 domains and in occlusion of the gp41 MPER.

It is worth emphasizing that antibody and peptide binding assays cannot differentiate between relevant conformations leading to fusion and off-pathway structures corresponding to an inactivated gp41. This notion is supported by the fact that antibodies against gp41 pre-bundles have been reported to react with gp41 outside the contact area between Env-expressing and target cells [117] or under conditions promoting gp41 inactivation, e.g., after sCD4 treatment in the absence of target cells [116,118]. This consideration highlights the advantages of functional assays (see below) that monitor the sensitivity of different stages of fusion to inhibitory peptides blocking 6HB formation. By definition, functional assays monitor the conformational status of Env trimers that participate in productive fusion.

#### Functional dissection of fusion intermediates

A powerful approach to elucidate the mechanism of HIV-1 Env-induced membrane merger involves dissection of individual steps of cell-cell [115,118,121-124,129-131] and virus-cell fusion [29,48,49]. This strategy is based upon capturing distinct intermediate stages of fusion and examining their resistance to inhibitors that target different steps of this reaction. As discussed above, the HR1 and HR2 domains are not exposed on a native gp41 or on the final 6HB structure [132], but these domains are available on pre-bundles formed upon interactions with receptors and/or coreceptors [122,126-128,130,133]. The formation of gp41 pre-bundles has been indirectly demonstrated by the gain-of-function experiments using the gp41-derived inhibitory peptides. This approach is based upon the addition of inhibitory peptides at distinct intermediates stages and assessing the peptide-gp41 binding by washing off the unbound peptide and restoring optimal conditions [121,123,124,129,130]. If this protocol attenuates the fusion activity, the complementary HR domains must have been exposed at a given intermediate stage. Conversely, the transition of gp41 pre-bundles to 6HBs can be detected using a loss-of-peptide-function assay (see below).

HIV-1 Env-mediated fusion is a steep function of temperature and is blocked at temperatures below a threshold value around 18–23°C, depending on the viral strain and expression levels of Env, receptors and coreceptors [122,124,134,135]. Prolonged (several hours) pre-incubation of Env-expressing and target cells at sub-threshold temperature results in formation of the temperature-arrested stage, TAS [130]. As evidenced by the resistance to inhibitors of CD4 and coreceptor binding, the majority of functionally active Env form ternary complexes with receptors and coreceptors at TAS without promoting hemifusion or fusion [124]. Thus, formation of ternary gp120-CD4-coreceptor complexes can be readily isolated from the subsequent restructuring of gp41 that leads to a membrane merger. Even though fusion does not occur at TAS, the gp41 HR1 and HR2 domains are exposed at this stage, as evidenced by sensitivity of fusion to C- and N-peptides added and washed off prior to raising the temperature [122,130].

To identify the most advanced functional conformation of gp41 upstream of membrane merger, the fusion must be captured at physiological temperature. Disparate biological fusion reactions converge to a common lipid-dependent stage that can be reversibly blocked by incorporating lyso-lipids into the contacting leaflets of fusing membranes (reviewed in [136]). Lyso-lipids (e.g., lyso-phosphatidylcholine) inhibit fusion by disfavoring the lipid monolayer bending into a stalk intermediate (Fig. 1A). By taking advantage of the ability of lyso-lipids to reversibly block fusion upstream of membrane merger, HIV-1 Env-induced fusion has been captured at permissive temperature [121,130]. The C- and N-peptides added at this intermediate stage termed a lipid-arrested stage (LAS) inhibited the fusion that would have otherwise occurred upon the removal of lyso-lipids. This finding demonstrates that gp41 does not form 6HBs prior to membrane merger even at optimal temperature.

**Figure 2:**
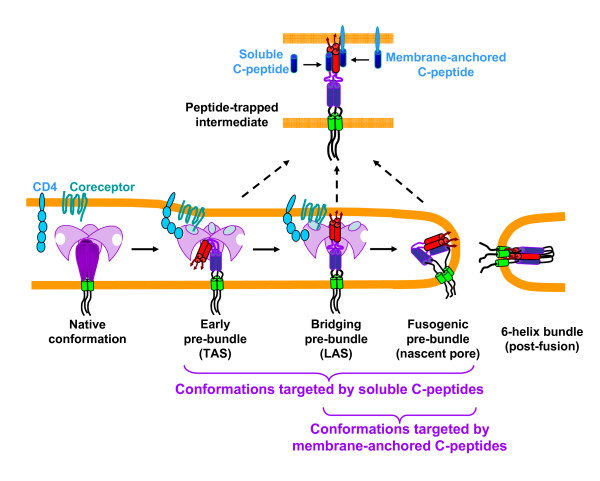
**Intermediate steps of HIV-1 Env-induced fusion progressing through early (TAS, temperature-arrested stage), bridging (LAS, lipid-arrested stage) and fusogenic pre-bundles toward 6-helix bundles that form after opening of a fusion pore**. Membrane-anchored C-peptides capture the extended conformation of gp41.

The conformational status of gp41 at TAS and LAS upstream of membrane merger has been further characterized by employing C-peptides anchored to the target membrane through a short linker and a single transmembrane domain [137,138]. These spatially and orientationally constrained C-peptides were used to capture a subset of gp41 pre-bundles that directly engaged the target membrane [129]. These spatial constraints conferred selectivity to the anchored C-peptides permitting their interactions only with a subset of gp41 pre-bundles that inserted their fusion peptides into the target membrane (Fig. 2). Compared to control experiments when fusion was not interrupted, the inhibitory activity of membrane-anchored peptides observed upon restoring optimal conditions was greatly enhanced after creating LAS, but not after TAS. This implies that gp41 conformations captured at fusion-permissive temperature directly engage the target membrane, permitting ample time for binding of anchored C-peptides and thereby potentiating their inhibitory effect. The lack of direct interactions between gp41 and target membrane at sub-threshold temperature is supported by the lack of gp41 labeling at TAS by photoactivatable hydrophobic probe incorporated into target cells [139].

Considering the extreme stability of gp41 6HBs in solution [140,141], these structures should not readily regress back to pre-bundles and thus should not interact with soluble C- or N-peptides [133]. Therefore, the acquisition of resistance to soluble inhibitory peptides added at an advanced intermediate stage should herald the formation of a requisite number of 6HBs at the fusion site. This strategy revealed that gp41 folding into the 6HB is completed after (but not before) the opening of a fusion pore [123]. Briefly, the addition of inhibitory peptides resulted in the quick and irreversible collapse of nascent pores arrested by lowering the temperature immediately after their formation. Thus, small pores are formed before a requisite number of gp41 completes refolding into the 6HB. This finding demonstrates that, contrary to a common perception, fusion pores are formed by gp41 pre-bundles, whereas 6HBs may play a role in stabilizing and perhaps expanding nascent pores. The sensitivity of nascent pores to inhibitory peptides also implies that the fusogenic gp41 pre-bundles are reversible conformations and that fusion pores are energetically unfavorable structures, prone to closing without the supporting fusogenic proteins. In summary, studies of the resistance of various fusion intermediates to soluble and membrane-anchored C-peptides led to identification of three distinct gp41 pre-bundle intermediates – early, bridging and fusogenic pre-bundles (Fig. 2) [123,129,130].

## The role of 6HB formation in fusion induced by other class I viral proteins

It is worth pointing out that 6HBs are only a part of the trimeric hairpin motif of class I proteins. There is evidence that regions outside the HR1/HR2 domains play a role in fusion. For instance, the membrane-proximal external region (MPER) and residues adjacent to the fusion peptide are essential for the formation and growth of a fusion pore mediated by HIV-1 Env and influenza hemagglutinin [78,142,143]. Interestingly, ASLV Env appears to form 6HBs at low pH prior to membrane merger, as evidenced by resistance of fusion to the inhibitory HR2-derived peptide added at a lipid-arrested stage [144]. This finding suggests that, unlike the HIV-1 Env [123] and paramyxovirus F [145] glycoproteins, interactions between residues outside the ASLV heptad repeat domains are responsible for hemifusion and fusion. The degree of coupling between bundle formation and membrane merger may depend on the length and/or flexibility of a region between the HR2 and MSD. It thus appears that, in order to induce fusion, viral proteins must zipper completely and bring their membrane-anchored regions (MSDs and fusion peptides) into close proximity. Interactions between HR1 and HR2 domains within the 6HB may or may not provide the main driving force for a fully zippered structure. We and others [11,61] have hypothesized that fully assembled hairpins permit direct interactions between MSDs and fusion peptides, which may destabilize a hemifusion diaphragm and promote opening of a fusion pore (Fig. 1B).

## Pore growth and nucleocapsid delivery

Dilation of fusion pores to sizes that permit viral nucleocapsid delivery (~50 nm) is critical for infection, yet the mechanism of pore enlargement is not understood. Studies of influenza hemagglutinin and HIV Env-induced cell-cell fusion showed that nascent pores are reversible structures sustained by fusion proteins [123]. Several lines of evidence suggest that the reliance on energy provided by viral proteins increases as fusion progresses from hemifusion to pore opening and pore enlargement steps [78,84,123,146-150]. First, the GPI-anchored ectodomain of influenza hemagglutinin is capable of promoting hemifusion and, with much lower probability, small non-enlarging pores [148,151]. In other words, lipid mixing can be readily achieved by the ectodomain anchored to the external leaflet of a plasma membrane, whereas a full-length protein is required to form expanding pores. Second, complete fusion (content mixing) appears to require a greater density of active proteins compared to hemifusion (lipid mixing) [48,84,147,150]. Third, the number of cell pairs exhibiting lipid mixing is usually greater than those forming small fusion pores, and only a minor fraction of nascent pores enlarge [148,152]. These observations support the notion that formation, and especially dilation, of small pores is energetically unfavorable compared to hemifusion. Thus, a greater number of active fusion proteins is required to form and sustain functional pores.

**Figure 3:**
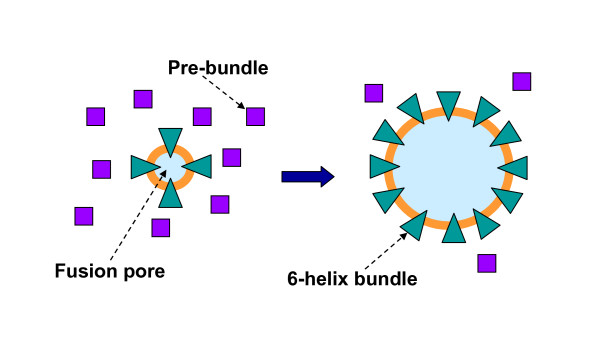
**The model for pore expansion via recruitment of fusion proteins (top view)**. Fusion proteins that require membrane continuity to complete their folding into a 6-helix bundle should accumulate at the perimeter of a fusion pore thereby promoting its enlargement.

The above considerations and several lines of functional evidence [20,153-156] indicate that successful fusion is achieved through the concerted action of several viral proteins. For those class I proteins that exhibit strict coupling between 6HB formation and membrane merger [123,130,157], pore growth could occur through recruiting additional proteins into its edge [123]. The ability to form the lowest energy 6HB structure at the pore perimeter, but not at sites of membrane apposition, would drive the pre-bundle incorporation into a nascent pore (Fig. 3). The limitation of this model is that it requires a large number of activated fusion proteins in the vicinity of a pore and is applicable only to proteins that cannot form 6HBs prior to membrane merger.

Recent work has challenged a common view that several proteins are required to form a functional fusion pore. Based on measurements of infectivity as a function of the ratio of the wild-type to a dominant-negative mutant of HIV-1 Env incorporated into virions, Yang and co-authors concluded that a single Env may mediate productive entry [32]. However, this conclusion is model-dependent. The more rigorous theoretical analysis of the above data yielded a greater number of HIV-1 Env (between 5 and 8) in a fusion complex [158,159]. Can a single viral protein store sufficient conformational energy to cause fusion? While estimates of the energy required for pore formation are available [160-162], the energy released upon refolding into a complete trimeric hairpin (including possible interactions between MSDs and fusion peptides) has not been determined. It is also not known how efficiently this conformational energy is utilized to restructure lipid bilayers. Regardless of the energy stored in fusion proteins, a single protein might not be able to exert a force to reshape and rupture fluid membranes. There is evidence that, in order to destabilize and merge two bilayers, fusion proteins must first form an oligomeric "fence" that restricts the lateral diffusion of lipids [84].

The controversy around the stoichiometry of fusion complexes suggests that perhaps this problem should be considered in a different context. Viruses often rely on cellular signaling and actin remodeling to enhance infection [163,164]. For instance, HIV Env-mediated signaling via CD4 and/or coreceptors has been implicated in productive entry [18,39,50,165-170] and Env-mediated fusion [131,165,168,171]. It is thus tempting to speculate that viruses may accomplish the formidable task of creating and expanding a fusion pore by hijacking the cellular machinery. In other words, viral proteins could utilize their conformational energy to promote hemifusion and to create a small pore while relying on a host cell to carry out the energetically costly step of pore dilation. For instance, VSV may undergo low pH-dependent fusion with intralumenal vesicles of early/intermediate endosomes and release its capsid into the cytosol via the constitutive "back-fusion" reaction between intralumenal vesicles and the limiting membrane of a late endosome [42]. However, this two-step fusion entry model for VSV has recently been challenged [172]. Thus, the role of cellular processes in the dilation of viral fusion pores has yet to be unambiguously determined.

The cytoskeleton may facilitate retrovirus entry not only by promoting receptor clustering on the cell surface [131,173-175] or transport of bound viruses along microvilli to the cell body [24], but also by augmenting the fusion and early post-fusion steps ([174,176] and references therein). The exploitation of cellular processes to drive the energetically costly step of pore dilation could explain the ability of a few (perhaps even a single [32,177]) retroviral Env to initiate infection. Once a hemifusion intermediate or a small fusion pore is formed, viral capsid delivery might be augmented by cytoskeleton rearrangements and/or by membrane trafficking machinery.

## Conclusion

Recent studies of viral fusogens revealed that structurally diverse proteins may have adopted a common "cast-and-fold" mechanism to merge membranes. Moreover, the general principles of viral fusion could be shared by proteins responsible for intracellular and developmental fusion [178,179]. This common mechanism is likely dictated by the physical properties of lipid bilayers and by the necessity to follow the least energy-costly membrane restructuring pathway leading to fusion without disrupting the membrane barrier function. While structures of the ectodomains or the core fragments of viral proteins showed that these proteins undergo major restructuring that culminates in formation of a trimeric hairpin, the actual refolding pathways remained conjectural. Functional studies demonstrated that viral fusion progresses through a number of distinct, reversible and increasingly unfavorable steps. The notion that formation, and especially enlargement of fusion pores, is an uphill process changes our views on how viral proteins may function. The increasing cost of forming and enlarging fusion pores indicates that viral fusogens should form oligomeric complexes capable of exerting an increasing force as fusion progresses to completion. In addition, viruses may rely on cellular machinery to enlarge fusion pores and release their capsid into the cytosol. Advances in understanding both the molecular details and unifying principles of viral protein-mediated fusion should help identify new targets for antiviral therapy and vaccine development.

## Abbreviations

6HB: six-helix bundle structure; ASLV: Avian Sarcoma and Leukosis Virus; Env: envelope glycoprotein; GPI: glycosylphosphatidylinositol; HR1 and HR2: helical heptad repeat 1 and 2 domains of class I viral fusion proteins; LAS: a lipid-arrested stage of fusion; MLV: Murine Leukemia Virus; MPER: membrane-proximal external domain of a fusion protein; MSD: membrane-spanning domain; SU and TM: surface and transmembrane subunits, respectively, of a fusion protein; TAS: a temperature-arrested stage of fusion; VSV: Vesicular Stomatitis Virus.

## Competing interests

The author declares that they have no competing interests.

## Acknowledgements

The author would like to thank Dr. Kosuke Miyauchi for critical reading of the manuscript and stimulating discussions. This work was supported by NIH R01 grants GM054787 and AI053668.
